# Abortion services during the COVID-19 pandemic: a systematic review

**DOI:** 10.1186/s12978-023-01582-3

**Published:** 2023-04-13

**Authors:** Kowsar Qaderi, Rasa Khodavirdilou, Mehri Kalhor, Bahar Morshed Behbahani, Maryam Keshavarz, Maryam Hassanzadeh Bashtian, Mahsa Dabir, Morvarid Irani, Elham Manouchehri, Maryam Farmahini Farahani, Manthar Ali Mallah, Ahmadreza Shamsabadi

**Affiliations:** 1 Midwifery Department, School of Nursing and Midwifery, Kermanshah University of Medical Sciences, Kermanshah, Iran; 2 Faculty of Advanced Medical Sciences, Tabriz University of Medical Sciences, Tabriz, Iran; 3 Department of Midwifery, School of Nursing and Midwifery, Shahid Beheshti University of Medical Sciences, Tehran, Iran; 4 Reproductive Health Department of Midwifery, School of Nursing and Midwifery, Shiraz University of Medical Sciences, Shiraz, Iran; 5 School of Nursing and Midwifery, Iran University of Medical Sciences, Tehran, Iran; 6 School of Medicine. North, Khorasan University of Medical Sciences, Bojnurd, Iran; 7 USERN Office, Kermanshah University of Medical Sciences, Kermanshah, Iran; 8 School of Nursing and Midwifery, Torbat Heydarieh University of Medical Sciences, Torbat Heydarieh, Iran; 9 Department of Midwifery, Mashhad Branch, Islamic Azad University, Mashhad, Iran; 10 Department of Midwifery, Faculty of Nursing and Midwifery, Tehran Medical Science, Islamic Azad University, Tehran, Iran; 11 College of Public Health, Zhengzhou University, 100 Kexue Ave, Zhongyuan District, Zhengzhou, 450001 China; 12 Department of Health Information Technology, Esfarayen Faculty of Medical Science, Esfarayen, Iran

**Keywords:** Abortion, COVID-19, Telemedicine, Teleconsultation, Healthcare services, Systematic review

## Abstract

Evidence suggests that COVID-19 may impair access to sexual and reproductive health services and safe abortion. The purpose of this systematic review was investigating the changes of abortion services in the COVID-19 pandemic era. We searched PubMed, Web of Science and Scopus for relevant studies published as of August 2021, using relevant keywords. RCT and non-original studies were excluded from the analysis and 17 studies of 151 included in our review. Requests to access medication abortion by telemedicine and demand for self-managed abortion were the main findings of identified studies. Women requested an abortion earlier in their pregnancy, and were satisfied with tele-abortion care due to its flexibility, and ongoing telephone support. Presenting telemedicine services without ultrasound has also been reported. Visits to clinics were reduced based on the severity of the restrictions, and abortion clinics had less revenue, more costs, and more changes in the work style of their healthcare providers. Telemedicine was reported safe, effective, acceptable, and empowering for women. Reasons for using tele-abortion were privacy, secrecy, comfort, using modern contraception, employing of women, distance from clinics, travel restrictions, lockdowns, fear of COVID-19, and political reasons (abortion prohibition). Complications of women using tele-abortion were pain, lack of psychological support, bleeding, and need to blood transfusions. The results of this study showed that using telemedicine and teleconsultations for medical abortion in the pandemic conditions may be extended after pandemic. Findings can be used by reproductive healthcare providers and policy makers to address the complications of abortion services.

*Trail registration* This study is registered in PROSPERO with number CRD42021279042

**Supplementary Information:**

The online version contains supplementary material available at 10.1186/s12978-023-01582-3.

## Abstract (summary)

COVID-19 pandemic shocks the international community, especially health policymakers around the world. The most important consequence of this outbreak has been direct and indirect impacts on health service provisions in all parts of the health system, including sexual and reproductive health services. We reviewed numerous studies investigating healthcare related to abortion in the pandemic era that showed women had more requests to access medical abortion, more than surgical. They preferred self-managed abortion process by telemedicine. Presenting telemedicine services without ultrasound has also been reported. Visits to clinics were reduced, and this decrease was reported based on the severity of the restrictions. Abortion clinics had reduced revenue, increased costs, and changed work style of their healthcare providers. Reasons for using telemedicine were fear of COVID-19, travel restrictions, lockdowns, more privacy, secrecy, and comfort. Telemedicine was reported safe, effective, acceptable, satisfying, and empowering for women. Maternal complications using tele-abortion were pain, bleeding, and need to blood transfusions. These findings can be used by policy makers and reproductive healthcare providers to address the complications of abortion management.

**Supplementary Information:**

The online version contains supplementary material available at 10.1186/s12978-023-01582-3.

## Background

COVID-19 pandemic has put a lot of pressure on the health systems of countries around the world [1, 2]. The burden of infection and the high mortality and morbidity rates have led health systems to do their utmost to combat it. The national health services of the affected countries faced lack of funding, inadequate finance, deprivation of human and technical resources, and rigid and fragmented health policy-making [1, 3].

The coronavirus pandemic, directly and indirectly, has affected health service provisions in all parts of the health system, including reproductive health services such as maternity care, family planning, and sexual health [4, 5]. Coronavirus infection and its complications in mothers increased the need for special care in the obstetrics ward. Fear, stigma, misinformation, and socioeconomic factors including restrictions, lack of financial resources, reduced economic activity, and reduced government revenues indirectly affected the access to essential reproductive health services [4–6].

Reduction in access to and utilization of essential reproductive health services during the coronavirus pandemic increased the number of women who suffer from complications or die during pregnancy [7, 8]. An abortion, or termination of pregnancy, is a procedure to end a pregnancy. Abortion services include ending pregnancy either by taking medicines or having a surgical procedure. In addition, abortion services and stock-out of contraceptives to prevent unintended pregnancies are disrupted [7–9]. A 10% reduction in service coverage during reproductive age could result in the death of an additional 28,000 mothers, over 3.3 million unsafe abortions, and 15.4 million unintended pregnancies as family planning services face disruptions [8, 10]. Access to sexual health services and safe abortion reduced in many countries in COVID-19 pandemic lockdowns. This issue can increase the mortality of adolescent women and girls who are more vulnerable to unintended pregnancies than others [9].

Unsafe abortion is one of the most critical problems of reproductive health services, which is more common in middle and low-income countries. That is due to the lack of access to legal abortion services and financial resources [11, 12]. About 7 million women are admitted to hospitals in these countries every year due to the complications of unsafe abortion. Annually, about 4.7 to 13.2% of maternal deaths occur due to unsafe abortion, and the cost of management of the complications of unsafe abortion is estimated at US$ 553 million [12, 13].

Concerning the morbidities and high burden of unsafe abortions, in cases where safe abortion services are limited or are not available, people resort to using herbs or drugs or surgical procedures from unknown and often unsafe sources to terminate their pregnancies [14]. Some countries have recognized this risk during the COVID-19 pandemic and have allowed people with remote counseling or telemedicine to take some medications at home to avoid abortion with mentioned methods [14]. Therefore, some studies suggest that in these situations, health systems can use telemedicine, virtual and social networks to provide education and counselling on contraceptive methods or safe drugs for induced abortion to prevent the risk of unsafe abortion [15].

Global efforts were made in a crucial circumstance like this to quickly create safe and effective vaccinations. The first COVID-19 vaccination was ultimately authorized by the American Food and Drug Administration in August 2021 [16]. After immunization with this vaccine, fertility doesn’t appear to be impaired [16]. In these situations, it seems necessary to provide education and counselling about safe sexual health to prevent coronavirus infection, care before and after using contraceptive or abortion methods in the current pandemic. Despite numerous studies, some questions remain unanswered, including the impact of pandemic on the services for abortion and post-abortion and the strategies should the health systems adapt to manage abortions in the current pandemic. So far, no study has integrated all the strategies and practical approaches to administering this issue. Therefore, in this study, we intend to systematically review the studies investigating management of health services to abortions during the COVID-19 pandemic.

## Methods

This study is a systematic review of abortion services during the COVID-19 pandemic. With the intention of reliability and authenticity of the results, this report adhered to the Preferred Reporting Items for Systematic Reviews and Meta-Analyses (PRISMA) checklist. Also, this study is registered in PROSPERO with number CRD42021279042.

### Data sources

We searched comprehensively the online databases of PubMed, Web of Science, and Scopus for relevant studies which were published in English from December 2019 to August 2021 (see Additional file 1).

### Search strategy

The search strategy of the present study was organized in collaboration with two members of the research team. An electronic search was performed in each database based on the following keywords: abortion, miscarriage, feticide, SARS-CoV-2, Coronavirus, COVID-19. The complete search strategy is as follows:

Strategy search:

A. COVID-19 OR SARS-CoV-2 OR Corona virus
B. Abortion OR miscarriage OR abort OR feticide OR “pregnant termination”
C. [A] AND [B]

### Eligibility criteria

Retrieved studies should meet the following criteria to be included in this study.

- The original studies investigated abortion services during the COVID-19 pandemic
- The studies published from the beginning of the COVID-19 to August 2021

The articles which had at least one of the following criteria were excluded:

 − Non-original articles, including reviews, case reports, clinical trial protocol, and editorials
 − Articles without obtainable full texts, abstract papers, and conference abstracts
 − Non-English language

### Data retrieval

The EndNote software was used to organize articles of the systematic review. Search results from reviewed databases composed in a single EndNote library and duplicate records removed.

### Data screening

**Fig. 1:**
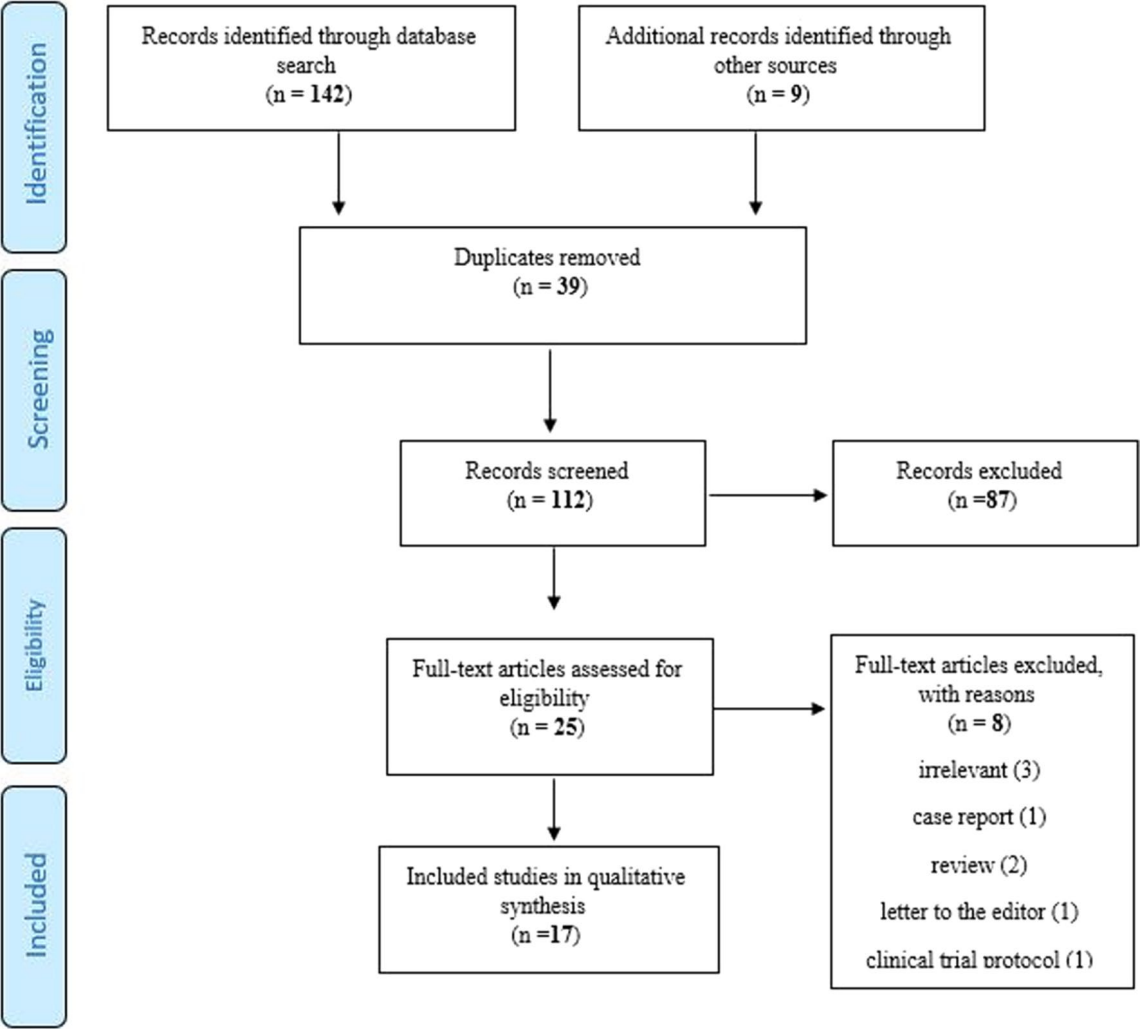
Prisma flowchart

Two research team members independently screen titles and abstracts of retrieved studies to determine if they meet inclusion and exclusion criteria. The process of study selection is shown in Fig. 1.

### Data extraction

This study extracted variables included the first author, year, type of evidence/ study, country, participants (number), age, abortion services, satisfaction, factors related to abortion services, maternal outcome, and other findings. Three authors independently extracted outcome data using the standardized table. Two members of the research team designed these specifications on the table. In order to exclude any duplications, the selected articles were surveyed by other researchers once again.

### Quality assessment

Two independent members of the research team assessed the quality of the cross-sectional and cohort studies by New Castle-Ottawa Scale (NOS), any disagreement was resolved by a third author, and the consensus was achieved.

## Results

### Selection and characteristics of included studies

The study selection process is shown in Fig. 1. One hundred fifty-one records were identified through the database and reference lists of articles. After removing duplicated records, 112 records remained; finally, 25 full-text articles were assessed for eligibility and seventeen articles have been included: Cross-sectional [17–19, 32, 33], prospective [20, 23, 34], retrospective [23, 24] cohort, qualitative [20, 25], mixed-method [21, 22], descriptive [26–30] studies and a newspaper [29]. Included studies have been conducted in USA, France, Belgium, UK, Scotland, Mexico, Columbia, Nepal, and eight European countries, as showed in Table 1.

**Table 1:** Characteristics of included studies and their main finding

ID	First author (reference)	Type of study	Country	Participants No.	Age	Abortion management (preferences and demands)	Satisfaction	Factors related to abortion management	Maternal outcome	Other findings	No. score
1	Aiken et al. [30]	Descriptive	8 European countries	3915 pregnant women	–	Increases in requests to access to medical abortion by telemedicine and demand for self-managed medical abortion	–	travel restrictions	–	Five countries showed significant increases in requests to Women on Web (WoW), ranging from 28% in Northern Ireland to 139% in Portugal	8
2	Aiken et al. [28]	Descriptive	USA	49,935 pregnant women (Up to 10 weeks)	–	27% increase in the rate of requests for self-managed abortion and online telemedicine Shifting in demand from in-clinic to self-managed abortion	–	lockdowns	–	Eleven states showed significant increases in requests, ranging from 22% in Ohio to 94% in Texas Increases in requests in states with the longest lasting restrictions	7
3	Atay et al. [22]	mixed-methods (Cross-sectional-Content Analysis)	France	809 Pregnant women 5 weeks <	Median 29 (11)	Demand for at-home medical abortion via teleconsultation	–	lockdowns	–	The most frequent reasons to choose telemedicine abortion was privacy (38.3%) secrecy (46.2%) and comfort (34.9%)	8
4	Aryal et al. [17]	Cross-sectional	Nepal	52 pregnant women (9.5- 7.5 weeks)	24.67 ± 4.08	Demand for SAS (Safe Abortion Services) decreased in 47.1% Individuals came at a later GA with a mean of9.5 weeks compared to 7.5 weeks. more women favorite medical abortion compared to surgical abortion	No significant difference in satisfaction towards services in lockdown and after it (p = 0.69)	Fear of COVID-19		19.2% individuals wanted termination of pregnancy in line for to fear of COVID-19	7
5	Boydell et al. [20]	Qualitative (Thematic analysis)	Scotland	20 pregnant women (Up to 12 weeks)	18–39	Expansion of a direct-to-patient telemedicine medical abortion/the quality of abortion care was improved in telemedicine service due to access, comfort, flexibility, and ongoing telephone support	Women accept telemedicine medical abortion at home	–	–	–	–
6	Chong et al. [26]	Descriptive	USA	1356 pregnant women (6 weeks)	15–47		85% was very satisfactory with TelAbortion	–	Transfusions (0.4%)	TelAbortion service was safe, effective, and acceptable	6
7	De Kort et al. [31]	Descriptive	Belgium	4 abortion centres	–	The number of applications for abortion in the clinic decreased. Individuals request an abortion earlier in their pregnancy	–	Individuals using modern contraception and in paid employing	Negative impact on the psychological support	Individuals using modern contraception and employing in paid had more reduced abortion requests in clinic	6-
8	De Kort et al. [25]	Qualitative (Phenomenological, abortion centre staff experiences)	Belgium	7 psychosocial staff members and 3 doctors	–	A general drop in abortion requests and procedures in abortion center. Technical and medical quality of abortions did not decline during the lockdown	–	–	–	People were more likely to request an abortion earlier in their pregnancy. Staff reported feeling stressed, tired, and frustrated	–
9	Gibelin et al. [24]	Retrospective	France	124 health workers performing abortions	–	The majority of abortion providers (76.6%) approved and followed medical abortion at home between 7–9W and 61.7% offered telemedicine for medical abortion at home	–	–	Bleeding, pain, hemorrhagic abortion	The French National Health Agency has urgently recommended telemedicine and consultations for medical abortion at home for 7–9 weeks pregnancies. This measure may be extended after pandemic	6
10	Godfrey et al. [32]	Cross-sectional	USA	534 pregnant women (10 weeks)	14–50	Direct delivery of medication abortion, online counseling, and care service from Aid Access	-	Location and distance from hospital	–	-71% lived in urban areas -24% lived in high Social Vulnerability Index (SVI) -26% living in medium–low SVI	7
11	Karlin et al. [33]	Prospective (interview and survey)	USA	40 abortion providers	–	Examining the change in the way clinics work and attitudes about self -sourced medication Telemedicine management abortion changes to the using of self-sourced medication	Believe about self-sourced medication abortion: Safe, effective, and empowering (50%) Ambivalent but safe and valid (45%) Unsafe (5%)	–	-	Another abortion protocol that clinics had was increasing gestational age limit to more than half After Covid 19, the need for in-clinic evaluation decreased and women find ultrasound less necessary before an abortion (decrease to 50%) or confirm pregnancy (decrease to 61%)	7
12	Kerestes et al. [23]	Retrospective cohort	USA	334 pregnant women (11 weeks) who had medication abortion	–	Success rate of abortion medication through telemedicine was 96.8, 97.1, and 93.6% for the clinic pickup, mail, and in person visit respectively. The effect of the medication abortion without surgical intervention was 95.8%	Telemedicine is satisfactory	–	Transferring emergency room (11), Blood transfusion (2), Receiving additional misoprostol (4)	149 patients received telemedicine with in-person pickup of medications, 75 patients via telemedicine with medications mailed, and 110 patients via traditional in person visits Using of medication abortion and present telemedicine service without ultrasound	8
13	LaRoche et al. [21]	Mixed method study	USA	711	48.3	Using medication abortion and telemedicine. people’s opinions about telemedicine to medication abortion	Using tele-medical abortion: agree (44%), disagree (35%), uncertain (21%)	Political reasons, when the life of the fetus begins, Safety	–		–
14	Romanis and Parsons [29]	Newspaper’s article	UK	–	–	Telemedicine services for early medication abortion (TEMA) minimize one’s contact with others	TEMA is safe, effective and satisfying	Distance from the nearest clinic	–	Somegovernments have banned abortion under any circumstances	–
15	Reynolds-Wright et al. [34]	prospective observational cohort	UK	663 pregnant women (12 weeks)	27.6	Using medical abortion at home with telemedicine services without routine ultrasound	83%: very acceptable 3.6: somewhat acceptable	–	Bleeding 2.4% Pain 2%	98% of women had a complete abortion, (0.8%) an ongoing pregnancy and (0.6%) an incomplete abortion	8
16	Andersen et al. [18]	Cross-sectional	USA and Columbia	317 clinics	–	Abortion restrictions have reduced the number of visits to abortion clinics by 32%	–	–	–	Areas where abortion is prohibited have an additional 23% reduction to visit the clinic	7
17	Marquez-Padilla et al. [19]	Cross-sectional	Mexico	Mexico City’s public abortion program data	–	The impact of stay-at-home orders on abortion	–	–	–	Stay at home can reduce abortions by at least 25%	7

### COVID-19 and abortion

The results showed that during the COVID -19 pandemic, requests for access to medication abortion by telemedicine and demand for self-managed medication abortion had been increased [20–24, 26, 28, 29, 34–36]. In contrast, the number of abortion requests and procedures in the abortion centers were generally dropped [31]. It was more significant in the most severe and longest-lasting lockdowns [28]. In another report, the number of visits to abortion clinics has been reduced by 32%, with an additional 23% reduction in areas where abortion is prohibited [18]. Travel restrictions [32], lockdowns [22, 27, 28], and fear of COVID-19 [17] were among reasons to choose telemedicine abortion. Request for telemedicine abortion was reported based on location and distance from the hospital [32].

### Satisfaction in telemedicine service

Numerous studies described tele-abortion safe, effective [20, 29, 32], very acceptable [20, 32, 34], and satisfying for women [23, 26, 29]. More individuals preferred medical abortion to surgical abortion [17]. In one study, the most frequent reasons to choose telemedicine abortion were privacy (38.3%), secrecy (46.2%), and comfort (34.9%) [22].

According to the results of a qualitative research, the quality of abortion care was improved in telemedicine services due to access, comfort, flexibility, and ongoing telephone support [20, 32]. It also reported that self-sourced medication abortion was safe, effective, and empowering for women [32, 33]. Another report showed no significant difference in satisfaction of services during and after lockdown (p = 0.690) [17].

### Complications and challenges of tele-abortion

The most reported complications of mothers were bleeding [24, 34], pain [24, 34], and need to blood transfusions (0.4%) [26]. The COVID-19 pandemic had created many challenges in abortion clinics, including changes in the work style of healthcare providers, increased costs, and reduced revenue, but care activities continued [37, 38]. Using medication abortion and present telemedicine services without ultrasound has also been reported [20, 23, 33].

## Discussion

Our results may indicate two different aspects. First, Increased rates of miscarriage throughout the pandemic may be due to the risk of COVID 19 during pregnancy, decreased access to prenatal care, or the financial downturn associated with the pandemic [39, 40]. Second, Decrease the rate of clinics appointments for abortion and increase the number of self-managed abortions, which can be due to fear of infection during the on-site visit or inability to go to the clinic due to disruption of the transportation system or childcare. We recognized higher stay-at-home behaviour levels with significant increases in requests in support of these probabilities. Studies have found that barriers to accessing the clinic, especially the cost of abortion, are reasons that individuals often cite. These barriers were reflected at the individual level at the state level, where the highest rates of applications were related to the residence in states with more restrictive abortion policies. There was also a correlation between the increase in the rate of requests in the counties, where the mean distance between nearest abortion clinics was longer, and the high proportion of the population living below the FPL [27], for example Texas, the state with the most prohibitive criteria, showed the greatest rise in requests, notwithstanding an almost low burden of COVID-19 [28]. International human rights law explicitly accredits the rights to sexual and reproductive health and autonomy of the body. These rights create a positive commitment by the government to provide information and services related to abortion and remove unnecessary medical barriers that eliminate practical access [41]. In times of crisis like pandemics, the international human rights commitments of states to respect, protect, and achieve the rights to health, life, and indiscrimination, among other rights, are not suspended. Steps to limit unsafe abortions and assure access to essential sexual and reproductive health services, such as abortion services, are key responsibilities of governments, even in emergencies. Achieving this main obligation demands the repeal of laws and procedures that criminalize, impede, or impair access to sexual and reproductive services, ensure public access to services, and limit unsafe abortions [42, 43]. Reaching these main obligations is vital and necessary in the time of COVID-19. Government responses that have promoted access to self-managed abortion are necessary steps to improve agreement with human rights obligations. Governments must fulfill similar proof-based and transformative solutions to guarantee abortion access for those who need a surgical abortion or those who do not have independence or basic support to offer self-managed abortion. States must more anticipate and deal with medical deficiencies due to interrupted supply chains. Other critical measures such as guaranteeing that telemedicine and other abortion services are possible to marginalized groups for free or at a low price. The results of a qualitative study showed that one of the common and positive experiences of maternal health care providers during the COVID-19 pandemic was the use of telemedicine capacity to care pregnant women that was beneficial in relieving their anxiety and breaking the chain of COVID-19 transmission [44]. However, telemedicine does not apply to all women and in all areas. Lack of adequate internet connection in some places prevents the widespread use of telemedicine [45].

## Limitations

This study has several limitations. First, even more than a year after the beginning of the COVID-19 epidemic, many aspects of reproductive health and abortion services are still unknown due to the lack of related articles. Second, existing studies sometimes report disparate material that cannot be discussed in the desired detail (because both our knowledge of the epidemic and its effects is rapidly increasing, and the results of the studies presented from different communities based on social and indigenous situations. Last, the present study was supposed to be done as a meta-analysis, but due to factors such as: the scarcity and heterogeneity of existing articles, the unknown nature of the disease, and its effects on reproductive health (including abortion), it was practically not possible.

## Suggestions

Based on the results and limitations of the study, in order to achieve more and better results, the following items are suggested:

1. Conducting studies with a wider range and more diverse variables regarding reproductive health and pregnancy.
2. Investigating and comparing the effects and complications of COVID-19 on reproductive health in different communities.
3. Investigating the effect of vaccination on the consequences of pregnancy and abortion (when we did this study, vaccination of pregnant women had not been done and we could not investigate the consequences of vaccination on pregnancy and abortion).

Overall, this study presents new findings on the impact of COVID-19 on aspects of abortion that can be used by reproductive health care providers to manage the complications of abortion.

## Conclusion

COVID-19 is a pandemic, which implies that global values need to be considered. It appears that countries with strict rules must revise their abortion laws throughout pandemics to decrease the unsafe abortions rate and their complications. The COVID-19 emergency is urging states to extend their healthcare systems and review their health laws. Women could suffer urgent harm if the restricted law is not repealed. Evidence suggests that COVID-19 may impair reproductive health, directly or indirectly. Given the effects of the COVID-19 epidemic on reproductive health, the results of this study provide detailed information on the various aspects of abortion and how to manage it in pandemic conditions. The findings of this study can be used by reproductive health care providers and policy makers to address the complications of abortion management.

## Supplementary Information

**Additional file 1.** Search details.

## Data Availability

The authors expressed that all information provided in this article can be shared.

## Abbreviation

FPL: Federal poverty level
