# Supplementary material for: Abortion services during the COVID-19 pandemic: a systematic review
Source: Reprod Health. 2023 Apr 13;20:61. doi: 10.1186/s12978-023-01582-3 (PMC10098996; doi:10.1186/s12978-023-01582-3)
Supplement: Supplementary file 1 — Additional file 1. Search details. [file 12978_2023_1582_MOESM1_ESM.docx]

**Additional file**

**ISI =31**

**TITLE:**(abortion) *OR* **TITLE:** (miscarriage) *OR* **TITLE:** (abort) *OR* **TITLE:** (feticide) *OR* **TITLE:** (“pregnant termination”) AND **TITLE:** (COVID-19) *OR* **TITLE:** (COVID) *OR* **TITLE:** ("Corona virus") *OR* **TITLE:** (SARS-CoV-2)

**SCOPUS = 48**

## **( ( TITLE ( covid-19 )  OR  TITLE ( covid )  OR  TITLE ( sars-cov-2 )  OR  TITLE ( "Corona virus" ) ) )  AND  ( ( TITLE ( abortion )  OR  TITLE ( miscarriage )  OR  TITLE ( abort )  OR  TITLE ( feticide )  OR  TITLE ( "pregnant termination" ) ) )  AND  ( LIMIT-TO ( DOCTYPE ,  "ar" ) )**

**PUBMED = 63**

((((((abortion[Title]) OR miscarriage[Title]) OR abort[Title]) OR feticide[Title]) OR "pregnant termination"[Title])) AND ((((COVID-19[Title]) OR "Corona virus"[Title]) OR SARS-CoV-2[Title]) OR COVID[Title])
